# Supplementary material for: Predicting visual function by interpreting a neuronal wiring diagram
Source: Nature. 2024 Oct 2;634(8032):113–23. doi: 10.1038/s41586-024-07953-5 (PMC11446822; doi:10.1038/s41586-024-07953-5)
Supplement: Supplementary file 6 — CRF and ERF predictions for individual TmY4 and TmY9 cells. Analogous to Supplementary Data 3, but for TmY target types. Shown are the top four monosynaptic pathways, the strongest pathway passing through each of the top ten intermediary types (ranking from Extended Data Fig. 7), and the trisynaptic pathway Tm1–TmY–Dm3–TmY (see the section entitled Prediction of spatial normalization). [file 41586_2024_7953_MOESM6_ESM.zip › DataS4/TmY9qΓèÑ/720575940628009577.pdf]

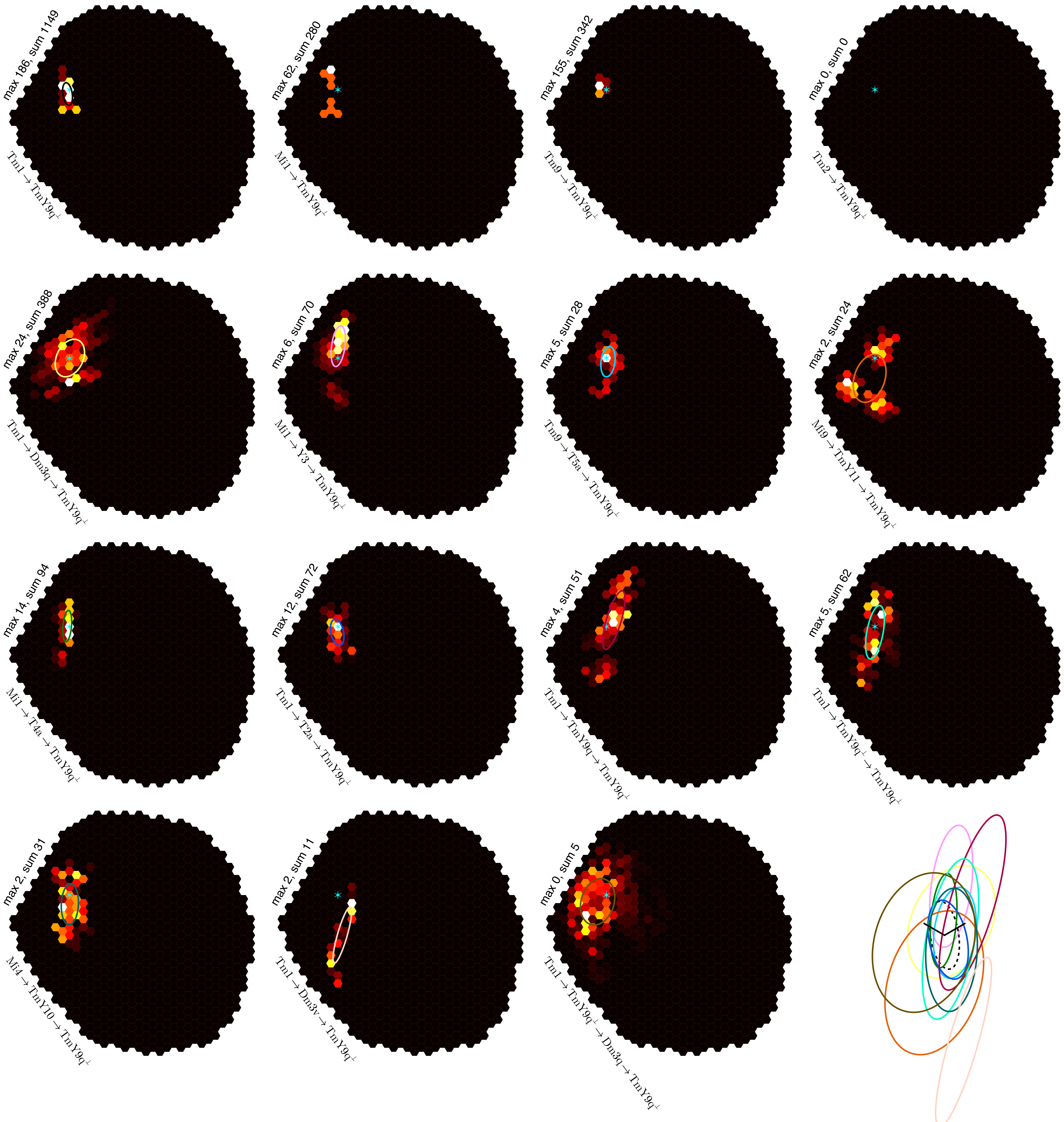
max 186, sum 1149
Tm1 → TmY9q⊥
max 62, sum 280
Mi1 → TmY9q⊥
max 155, sum 342
Tm9 → TmY9q⊥
max 0, sum 0
Tm2 → TmY9q⊥
max 24, sum 388
Tm1 → Dm3q → TmY9q⊥
max 6, sum 70
Mi1 → Y3 → TmY9q⊥
max 5, sum 28
Tm9 → T5a → TmY9q⊥
max 2, sum 24
Mi9 → TmY11 → TmY9q⊥
max 14, sum 94
Mi1 → T4a → TmY9q⊥
max 12, sum 72
Tm1 → T2a → TmY9q⊥
max 4, sum 51
Tm1 → TmY9q → TmY9q⊥
max 5, sum 62
Tm1 → TmY9q⊥ → TmY9q⊥
max 2, sum 31
Mi4 → TmY10 → TmY9q⊥
max 2, sum 11
Tm1 → Dm3v → TmY9q⊥
max 0, sum 5
Tm1 → TmY9q⊥ → Dm3q → TmY9q⊥
